# Intraspecific Trait Variation Driven by Plasticity and Ontogeny in *Hypochaeris radicata*

**DOI:** 10.1371/journal.pone.0109870

**Published:** 2014-10-21

**Authors:** Rachel M. Mitchell, Jonathan D. Bakker

**Affiliations:** 1 School of Environmental and Forest Sciences, University of Washington, Seattle, Washington, United States of America; 2 Smithsonian Environmental Research Center, Edgewater, Maryland, United States of America; University of Saskatchewan, Canada

## Abstract

The importance of intraspecific variation in plant functional traits for structuring communities and driving ecosystem processes is increasingly recognized, but mechanisms governing this variation are less studied. Variation could be due to adaptation to local conditions, plasticity in observed traits, or ontogeny. We investigated 1) whether abiotic stress caused individuals, maternal lines, and populations to exhibit trait convergence, 2) whether trait variation was primarily due to ecotypic differences or trait plasticity, and 3) whether traits varied with ontogeny. We sampled three populations of *Hypochaeris radicata* that differed significantly in rosette diameter and specific leaf area (SLA). We grew nine maternal lines from each population (27 lines total) under three greenhouse conditions: ambient conditions (control), 50% drought, or 80% shade. Plant diameter and relative chlorophyll content were measured throughout the experiment, and leaf shape, root∶shoot ratio, and SLA were measured after five weeks. We used hierarchical mixed-models and variance component analysis to quantify differences in treatment effects and the contributions of population of origin and maternal line to observed variation. Observed variation in plant traits was driven primarily by plasticity. Shade significantly influenced all measured traits. Plant diameter was the only trait that had a sizable proportion of trait variation (30%) explained by population of origin. There were significant ontogenetic differences for both plant diameter and relative chlorophyll content. When subjected to abiotic stress in the form of light or water limitation, *Hypochaeris radicata* exhibited significant trait variability. This variation was due primarily to trait plasticity, rather than to adaptation to local conditions, and also differed with ontogeny.

## Introduction

Recent and growing interest in plant functional traits has deepened our understanding of critical ecological mechanisms. For example, interspecific trait-based approaches have identified differing processes of community assembly across spatial scales [1] and in temperate and tropical ecosystems [2], shaped understanding of abiotic drivers such as altitude [3], [4], climate [5], [6], temperature [7], [8], and resource gradients [9] on community assembly and diversity, and identified global patterns of plant-trait expression [10]. In addition, trait-based approaches have provided a mechanistic understanding of correlations between biodiversity and ecosystem function [11]–[13].

Although trait-based approaches have improved our understanding of ecological mechanisms, the role of intraspecific trait variation - variation from individual to individual within a species - is less well understood. Intraspecific variation in plant functional traits can be large, though it has often been ignored or its contribution underestimated [14]–[16]. Intraspecific variation can structure communities [17] and influence ecosystem function [18]. For example, intraspecific variation in plant functional traits has been shown to structure communities along environmental gradients [19], to drive diversity in forest ecosystems [20], and to enhance community resistance to plant invasion [21].

Changes in intraspecific variation in plant functional traits have been observed in response to a wide range of environmental conditions (e.g., flooding [19], fire [22], drought [23] and temperature [24]), but few studies have examined the mechanisms controlling these changes (but see [25]–[27]). Two primary processes are hypothesized to drive variation in trait expression: adaptation to local conditions, creating distinct genetic ecotypes [28], and phenotypic plasticity in response to prevailing abiotic conditions [29]. Those studies that have investigated sources of intraspecific variation have, at times, found contrasting results, depending on the species and functional trait being investigated [30]–[32]. For example, Firn et al. found significant plasticity in specific leaf area (SLA; leaf area per unit mass) for an exotic, invasive grass but not for co-occurring native grasses [33]. In *Quercus suber*, genetically distinct populations displayed significantly different mean values for SLA, suggesting local adaptation to climate regimes, but populations also demonstrated high phenotypic plasticity [34]. In contrast, Robson et al. found that differences in SLA in *Fagus sylvatica* were primarily driven by genetic provenance [35].

If observed intraspecific trait variation is largely driven by the genetics of a given population, abiotic or biotic filters can selectively eliminate individuals with traits that exceed the bounds of the filter. For example, species or populations with low variability in traits related to drought stress (e.g., leaf size) may be unable to cope with reduced water availability under shifting climate. However, if variation in critical plant traits is largely a plastic response, independent of genetic diversity, even rare species may respond to filtering by altering their trait expression and thus persist in the community. However, plasticity is not without cost, as very plastic species may express phenotypes unsuited to abiotic filters, or experience costs associated with the trait of plasticity [36], [37].

In addition to being influenced by adaptation and plasticity, trait expression also changes due to ontogeny. Determining how traits vary ontogenetically is important because traits influence ecosystem function [11], and temporal differences in plant trait expression may facilitate species coexistence and biodiversity maintenance [38]. Smith *et al*. found that SLA decreased with age in *Populus tremuloides* [39], while Cornelissen et al. found poor correlation between greenhouse seedlings and field-measured adults for 90 woody plant species, indicating strong ontogenetic differences [40]. The effects of ontogeny can differ depending on the trait being measured. For example, wood density, but not leaf mass per unit area, was strongly affected by ontogeny in tropical *Nothofagus pumilio* [24]. Despite evidence of correlation between trait variability and ontogenetic state, this source of variability is rarely accounted for, and often explicitly avoided by use of standard collection protocols [41].

Examination of the mechanisms controlling intraspecific variation is confounded by the fact that variation is both adaptive and with cost [37], [42], [43]. One species, population within a species, or genetic lineage may be inherently more plastic than others for some traits. In a widely distributed species, some populations may exhibit greater plasticity in response to abiotic stress due to high environmental heterogeneity selecting for phenotypically plastic genotypes, or through genetic correlation to other traits that are under selection [44]. For example, Balaguer *et al*. [45] found that populations of *Quercus coccifera* growing in homogenous conditions displayed significantly less phenotypic plasticity in light-responsive traits than those growing in more heterogeneous conditions. Pratt & Mooney found a similar pattern for growth and flower production in *Artemisia californica* in response to precipitation variability [46].

We used *Hypochaeris radicata*, a globally widespread weed, as a model species for examining trait variation in terms of both trait means (average trait values) and trait dispersion (variability of trait values). *H. radicata* occurs in a number of ecosystems, from undisturbed grasslands to highly disturbed urban environments. Because it persists across such a wide variety of conditions, it likely exhibits considerable trait plasticity [47]. However, persistence under differing abiotic conditions may lead to distinct population-level trait differences through ecotypic adaptation or transgenerational plastic responses [14], [28], [38], [48].

We collected and germinated seeds from three populations, exposed the seedlings to abiotic stress (shade, drought) in the greenhouse, and measured a range of traits on each individual. We used these data to experimentally test three hypotheses. First, we hypothesized that abiotic stress would significantly impact trait expression for all populations, leading to a convergence in trait values for plants experiencing abiotic stress. Second, we hypothesized that population of origin and maternal line of origin, and not random variation between individuals, would explain the majority of observed trait variation that was not explained by abiotic conditions. Third, we hypothesized that over the lifetime of a plant, individuals, maternal lines, and populations within a treatment would become more similar in terms of both trait means and variances in order to accommodate stressful abiotic conditions.

## Materials and Methods

### Ethics Statement

No special permits were required for this research. Permission to conduct research at Glacial Heritage Preserve was granted by Thurston County. Permission to conduct research at Smith Prairie was granted by the non-profit owners, Pacific Rim Institute for Environmental Stewardship. Permission to collect samples at Union Bay Natural Area was granted by the University of Washington. This research did not include any populations of endangered or threatened species. Data used in this manuscript are available through the TRY database and can be accessed by submitting a data request at http://www.try-db.org.

### Study Species

*Hypochaeris radicata* is a short-lived perennial herb species common in intact and degraded grassland ecosystems around the globe, including Europe, North America, Australia, New Zealand and Japan. It forms basal rosettes with oblong-lanceolate leaves and produces several upright, branching flower stalks during a long flowering period – June through October in the Pacific Northwest of the USA. *H. radicata* is self-incompatible [49], [50] and its seeds are wind dispersed up to a few hundred meters [51].

### Experimental Design

**Figure 1:**
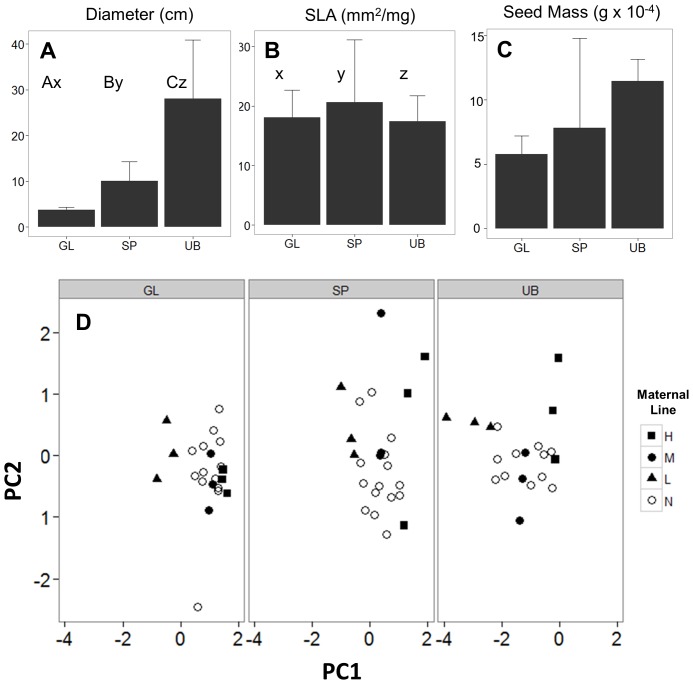
Mean (+ SD) of field-based measurements of three traits for the populations of origin (GL: Glacial Heritage; SP: Smith Prairie; UB: Union Bay). Significant PERMANOVA results (α = 0.05) are indicated by different uppercase letters and significant PERMDISP results are indicated by different lowercase letters. D) Results of a Principal Components Analysis of the three traits measured on individuals from each population. Populations were analyzed together, but are shown in separate graphs for clarity. PC1 explained 49% of observed variation. Filled symbols indicate maternal “high” (H), “medium” (M) and “low” (L) maternal lines used in the greenhouse experiment, while open symbols (N) indicate maternal lines measured in the field but not included in the experiment.

Three populations (Glacial Heritage (GL), Smith Prairie (SP), and Union Bay Natural Area (UB)) spanning a 200 km range in Washington state, USA, were sampled for this experiment. Populations occurred in exotic-dominated grasslands, but experienced different abiotic conditions in terms of annual precipitation, soil type, and land-use history (Table S1). Specific leaf area and rosette diameter were measured on 20–25 plants (maternal lines) per population, and one mature seed-head was collected from each plant. Measured plants were at least 3 m apart to prevent sampling from closely related individuals. Previous analyses detected significant differences in trait variability for SLA and rosette diameter [16]; follow-up analyses using PermDISP and PERMANOVA (see “Statistical Analysis”, below) indicated significant differences in trait dispersion and mean values for diameter (Fig. 1A, *P*<0.0001) and significantly different dispersion for SLA (Fig. 1B, *P* = 0.05) but no significant differences in seed mass (Fig. 1C).

The single seed head collected per maternal line did not contain enough seeds to allow individual maternal lines to be replicated in each abiotic treatment. Instead, maternal lines were subjected to Principal Components Analysis (PCA), and the first principal component (PC1) was used to select maternal lines spanning the range of traits expressed in each population. Thus, for each population, the three highest, three most central, and three lowest maternal lines (hereafter referred to as “high”, “central”, and “low”) along PC1, which explained 49% of the variation, were identified (Fig. 1D). One maternal line from each group was then randomly assigned to an abiotic treatment (Figure S1).

Seeds from the selected maternal lines (3 populations ×9 maternal lines/population) were cold-stratified for 10 days and germinated at 25°C. Twenty germinants per maternal line (540 total) were planted individually into 164 ml “conetainers” (Stuewe & Sons, Oregon, USA) filled with Sunshine Mix #3 potting soil (Sun Gro Horticulture, Massachusetts, USA). Germinants were allowed to acclimate for seven days before treatments were applied.

Plants were grown in the greenhouse under one of three treatments: ambient conditions, 80% shade (80% of light is blocked), and drought conditions (50% of winter precipitation). The shade and drought treatment levels were selected to mimic marginal conditions where *H. radicata* is known to survive. Beginning on day seven, the high, low and central maternal lines were randomly assigned to the three treatments, and individuals were randomly arranged on the bench to account for edge effects. The ambient and shade treatments received weekly top-watering equivalent to average October-November rainfall across the three populations (53 ml per week per conetainer). The drought treatment received half as much (26 ml per week per conetainer) and visibly induced plant stress; individuals wilted between waterings. Each treatment received weekly 10-10-10 NPK fertilizer plus micronutrients per manufacturer specifications.

Mortality and rosette diameter (a measure of competitive ability similar to height [16]) were measured weekly, beginning on day seven. Relative chlorophyll content (correlated with photosynthesis and leaf life-span [52]) was measured weekly beginning on day 14 (plant leaves were too small on day seven for data collection) using a SPAD 502 plus chlorophyll meter (Spectrum Technologies, Illinois, USA). The length and width of the largest leaf on each individual were measured after five weeks to quantify whether leaves were more lanceolate or ovate, which can have consequences for a number of functions, including light interception [53]. Plants were destructively sampled after five weeks. One leaf per individual was harvested, scanned, dried at 60°C, and weighed using a Mettler AE 163 analytical scale (Mettler Toledo, Ohio, USA). One-sided leaf area was calculated from the scanned images using ImageJ version 1.45 (http://rsb.nih.gov/ij). SLA, which is correlated with resource acquisition and leaf life-span [54], [55], was calculated as leaf area (mm^2^) divided by leaf dry mass (mg). Above and belowground biomass were harvested separately, dried at 60°C, weighed, and used to calculate root∶shoot ratio, a measure of resource allocation.

### Statistical Analysis

Differences between trait means for the populations of origin measured in the field were quantified using PERMANOVA, a non-parametric permutational form of ANOVA [56]. Differences in trait dispersion were quantified using PermDISP, a permutational analog to the Levene's test [57]. These analyses were conducted using the PERMANOVA+ add-on to PRIMER-E [58], with Euclidean distances, type III sums of squares, and 9,999 permutations. Populations were considered to differ if *P*<0.05.

For plants grown under greenhouse conditions, measured traits were checked for normality, and corrective measures were taken when traits were non-normal. Correlation between dependent variables was checked and, when response variables were strongly correlated, only one representative variable was tested (the weakest correlation among tested variables was *r^2^*>0.65, see Fig S2). Five traits were selected for analysis: diameter, leaf shape (leaf length∶width ratio; larger numbers indicate more lanceolate leaves and lower numbers indicate more ovate leaves), root∶shoot ratio, SLA, and final relative chlorophyll content. SLA and diameter were log transformed to correct for non-normality and heteroscedacity.

We used hierarchical linear mixed effects models and variance component analysis to quantify the influence of abiotic treatment and ecotypic factors on observed traits. Population and maternal line were designated as random effects, with maternal line nested within populations. The variance attributable to population of origin, maternal line, and individual were expressed as proportions of the total random-effects variance. Abiotic treatment (ambient, shade, or drought) was treated as a fixed effect. Models were fit with the “lme4” package in R (version 2.14.2) and *P*-values were generated using the “languageR' package. Significant treatment effect was followed by pairwise comparisons using TukeyHSD through the “multcomp” package (version 1.2–19), with attention focused on the differences between the ambient treatment and each of the shade and drought treatments.

Ontogenetic changes were examined by analyzing changes over time in diameter and relative chlorophyll content. These traits were selected because they did not require repeated, destructive measurements. Individual plants were nested within maternal lines within populations in these models, and time was included as a fixed effect. The fixed effects (abiotic treatment and time) and their interaction were iteratively added, beginning with the null model (including only random effects) and selecting the best model using Akaike's Information Criterion (AIC). Significant treatment by time effects were followed by separate analyses of each date to quantify the variance attributable to population of origin, maternal line, and individuals.

## Results

### Response to Abiotic Stress

**Figure 2:**
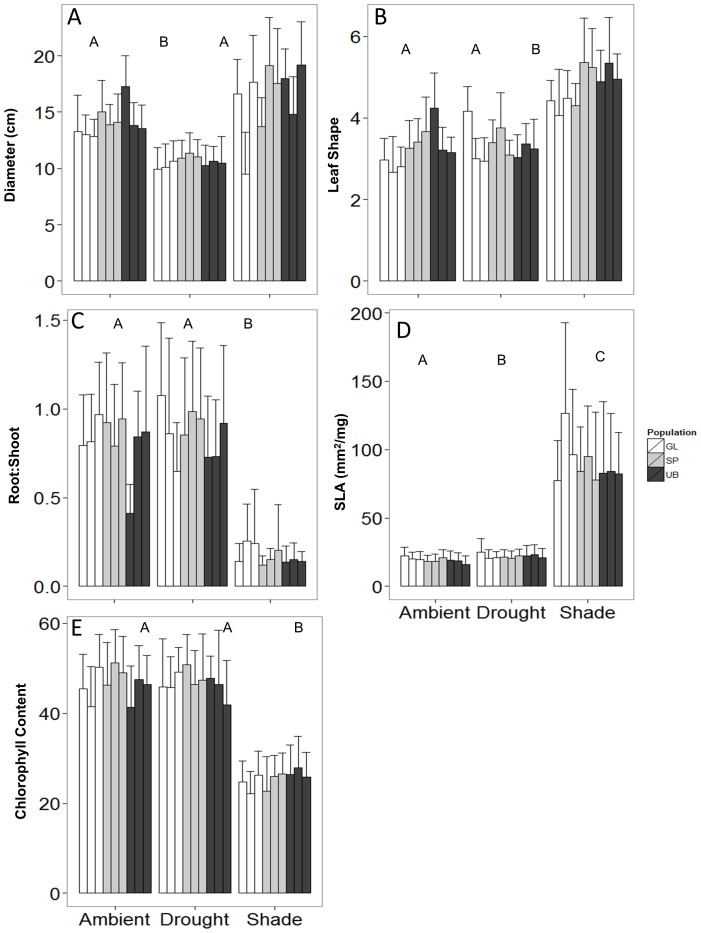
Mean (+ SD) values of A) final diameter, B) leaf shape (length∶width ratio), C) root∶shoot ratio, D) SLA, and E) final relative chlorophyll content for each maternal line (individual bar, arranged from low to high based on scores from PC1) in each population (unique color; defined in Table S1) for each treatment. Significant treatment differences (α = 0.05) in trait means are indicated by different letters.

**Figure 3:**
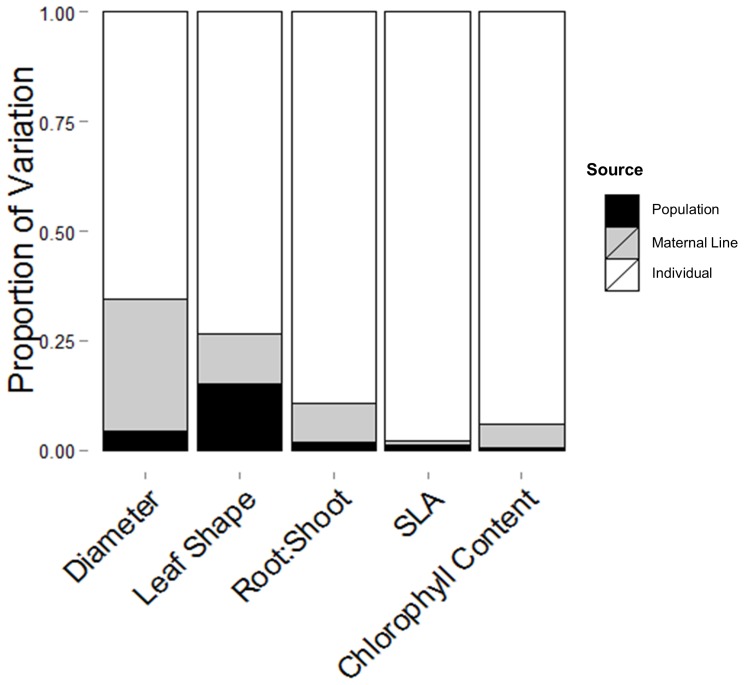
Proportion of total random effects variance explained by population of origin, maternal line, and individual plant (residual) for five measured traits.

Mortality was low, even for plants grown under abiotic stress: only 8 of 540 individuals died. However, the abiotic treatments affected plant trait expression (Fig. 2, See Table S2 for GLMM results). Mean final diameter was larger for plants in the shade treatment (16.4 cm) than the ambient treatment (14.1 cm), but smaller in the drought treatment (10.6 cm) (Fig. 2A). Plants in the shade treatment had significantly more lanceolate leaves (4.8) than those in the ambient (3.3) and drought treatments (3.3) (Fig. 2B), and also had significantly lower root∶shoot ratios (0.17 vs 0.82 and 0.86, respectively) (Fig. 2C). Both the shade and drought treatments increased mean SLA (88.2 and 22.1 mm^2^/mg, respectively) compared to those in the ambient treatment (19.4 mm^2^/mg) (Fig. 3D). Relative chlorophyll content was significantly lower for plants in the shade treatment (25.6 SPAD units) compared with those in the drought (46.8 units) or ambient treatments (46.7 SPAD units) (Fig. 2E).

### Contribution of Population and Maternal Line to Trait Variation

A surprisingly small amount of variation was explained by population of origin or maternal line for most measured traits (Fig. 3). Diameter was the only trait that showed an appreciable maternal contribution to observed variation; 30% of the total random effects variation was attributable to maternal lines, while 5% was attributable to populations (Fig. 3). Variation in leaf shape showed nearly equal contributions from population (15%) and maternal sources (12%). Visual inspection of the mean values for each maternal line showed no clear relationship with the maternal plant's score on PC1 (Fig. 1).

### Ontogenetic Effects

**Figure 4:**
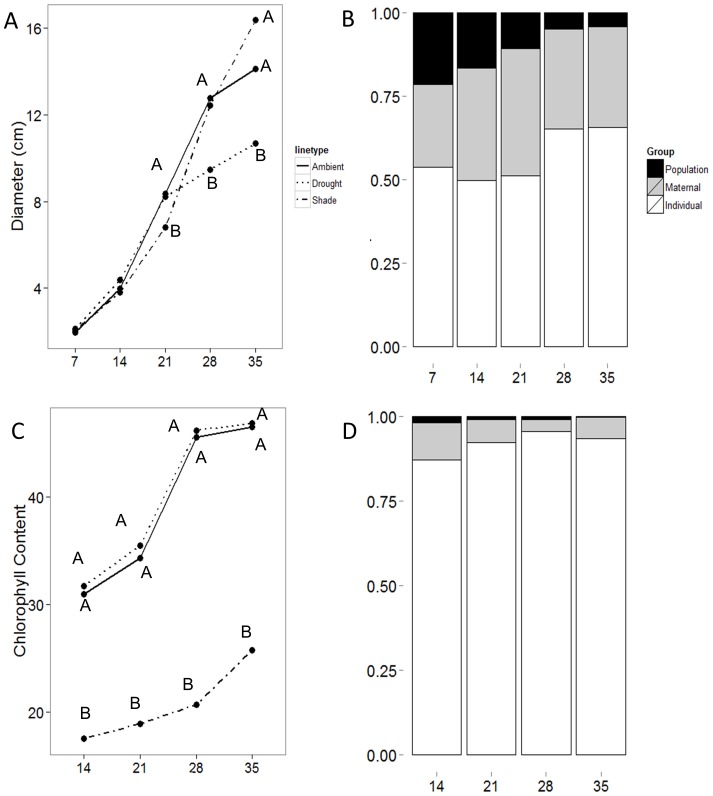
Change in average trait values in response to treatments (A and C; in each time period, letters indicate significantly different groups) and (B and D) the amount of variation attributed to population of origin, maternal line, and individual plant for diameter (top) and relative chlorophyll content (bottom).

There were significant interactions between treatment and time for both plant diameter and relative chlorophyll content (Fig. 4A and C). Treatment groups were indistinguishable on days 7 and 14. On day 21, plants in the shade treatment had significantly smaller diameter than those in the drought and ambient treatments (*P* = 0.03). On days 28 and 35, diameter in the drought treatment was significantly lower than the shade or ambient treatments (P≤0.0001; Fig 4A). Individuals accounted for an increasing amount of the variation in plant diameter, while variation between populations decreased over time (Fig. 4B). Variation between maternal lines remained fairly consistent.

Shading rapidly and strongly affected relative chlorophyll content, as indicated by significant treatment effects already on day 14, while drought did not (*P*<0.0001; Fig 4C). Variation between individuals increased between days 14 and 21, and remained high throughout the experiment (Fig. 4D). Variation between maternal lines and populations decreased over the course of the experiment.

## Discussion

### The roles of plasticity and adaptation in shaping trait expression

We set out to determine whether observed intraspecific variation was driven by plasticity or by adaptation to local abiotic conditions. Our results indicate that, for *H. radicata*, the observed trait variation is largely attributable to plasticity. Neither shading nor drought stress were strong enough to cause appreciable mortality, confirming that *H. radicata* can tolerate a very wide range of abiotic conditions. However, plants grown under these conditions exhibited markedly different functional traits. For example, SLA values previously reported for *H. radicata* individuals have ranged from 1.7–46.1 mm^2^/mg [16], [59], [60]; in this study, SLA ranged from 6.6–239.1 mm^2^/mg. Much of the increased range of SLA in this study was due to increases in response to shading (Fig. 2D), a response which has been demonstrated in the past [61]. The inclusion of shade-grown individuals, which have significantly higher SLA than sun-grown individuals, would dramatically shift the trait distribution for this species. Inclusion of such plants would also likely influence the overall trait distribution of the plant community, and may in turn impact estimations of critical ecosystem processes like litter production and nutrient cycling [13] through differing nutrient contributions and breakdown times in the decomposition pool [62]. Standard trait collection procedures [41], [63] focus trait measurements exclusively on sun-exposed plants and therefore do not account for the full trait variation of species that can tolerate varying levels of shade. The omission of individuals in partial or full-shade from trait measurements may hinder our understanding of trait mediated ecosystem processes and responses. We suggest it would be more appropriate to sample the full range of conditions experienced by a species.

The variance explained by population of origin and maternal line was surprisingly small for every measured trait. Only leaf shape and diameter had significant variation attributable to differences across populations and maternal lines respectively, and even this amount of variation (30%) was dwarfed by treatment effects. Virtually none of the observed variation could be attributed to population or maternal line for SLA (Fig. 3), confirming that this trait is highly plastic, a finding which supports previous analyses of this trait [61]. Furthermore, the position of each maternal line on PC1 had little correlation with the ranking of each maternal line in the greenhouse, suggesting that individuals within maternal lines exhibited significant variability and plasticity, and are not necessarily constrained by maternal genetics. The very small amount of variation attributable to population or maternal sources indicates that, for the traits measured in this experiment, plastic responses to prevailing conditions are the primary driver of observed variation.

The fact that all of the measured traits displayed significant plasticity has important implications for how traits are measured, and how they are incorporated into larger, trait-based models. Although some species are shade-intolerant, many species experience variable light conditions through interactions with neighboring plants in the community. Trait values based on fully sun-exposed leaves may inaccurately represent the trait contributions of such species, and thus may misrepresent community-level trait values, and the ecosystem functions dependent on those trait values. In addition, attempts to model species presence or response to abiotic change may be hindered by a reliance on trait means derived from sun-exposed individuals. If species, or populations within species, are variable and highly plastic for traits critical to survival, such taxa may be present in a wider range of habitat types, and be better able to cope with a wider range of conditions [64], [65] than expected from trait values measured on individuals growing in a subset of the taxa's possible growing conditions.

### The role of ontogeny in shaping trait expression

Variation in trait values over the lifespan of a plant is an additional, and often unaccounted for, source of intraspecific trait variability. This source of variation may be underappreciated as collection techniques typically focus on “mature” plants and tissues [41] yet maturity can be difficult to distinguish in the field. Because plants of differing ontogenies may appear “mature” in the field, intraspecific trait variation measured in field populations could be partially attributed to differing ontogeny between individuals, rather than to genetic or environmental conditions.

Furthermore, ontogenetic differences in trait variability may be important in the structure and function of communities. For example, ontogenetic trait differences may lead to temporal niche partitioning, facilitating biodiversity maintenance through limiting similarity [66]. Ontogenetic differences leading to higher palatability can also alter plant fitness by influencing interactions with herbivores and predators [67]. Furthermore, temporal variation in plant traits may exacerbate or mediate the impacts of abiotic filters. Young plants face similar pressures during establishment, and tend to exhibit similar trait values, in both this study, and others (e.g., [68]). This lack of trait variability in young plants may increase susceptibility to stress, while increased variability in older plants may buffer the population against abiotic change. Incorporating trait values of plants at different ontogenetic stages could provide a clearer understanding of community and population dynamics, and more accurate extrapolation to ecosystem processes and functions.

## Conclusion

Taken together, these results indicate that plasticity in response to abiotic conditions and ontogeny, and not adaptation and genetically distinct ecotypes, drive trait expression in *H. radicata*. However, the generality of this conclusion needs to be explored. *Hypochaeris radicata* is globally widespread, and thus may be inherently more plastic than species with restricted distributions [69], [70]. To determine whether plasticity is a characteristic trait of widespread or dominant species, this research should be replicated with other widespread species and with species from restricted ranges. In addition, further research should examine how sensitive these conclusions are to the identity of the trait being examined.

The treatments applied during this experiment represented strong abiotic filters for this species, but are within the range of conditions experienced by *H. radicata*. Studies involving light or moisture gradients could assess whether the importance of population of origin or plasticity changes depending on the strength of the abiotic filter, a question which was not addressed in this study. Studies could also examine other abiotic filters, such as soil fertility. Finally, the additive and interactive effects of multiple abiotic filters on trait expression could be tested in both field and greenhouse settings.

Despite growing interest in understanding and incorporating intraspecific variation in plant functional traits into larger community- and ecosystem-level studies, the factors driving observed variation remain poorly understood. Our results suggest that observed intraspecific variation is largely driven by trait plasticity rather than genetic factors. We also found strong evidence of variation in plant traits due to ontogeny, a source of trait variation that is often ignored.

## Supporting Information

Figure S1 **Diagram illustrating selection of populations and maternal lines.**
(DOCX)

Figure S2 **Correlation Matrix of Traits.**
(DOCX)

Table S1 **Table of Abiotic Conditions at Sample Locations.**
(DOCX)

Table S2 **Results of General Linear Mixed Models.**
(DOCX)
