# Supplementary figures and images for: Intraspecific Trait Variation Driven by Plasticity and Ontogeny in Hypochaeris radicata
Source: PLoS One. 2014 Oct 21;9(10):e109870. doi: 10.1371/journal.pone.0109870 (PMC4204820; doi:10.1371/journal.pone.0109870)

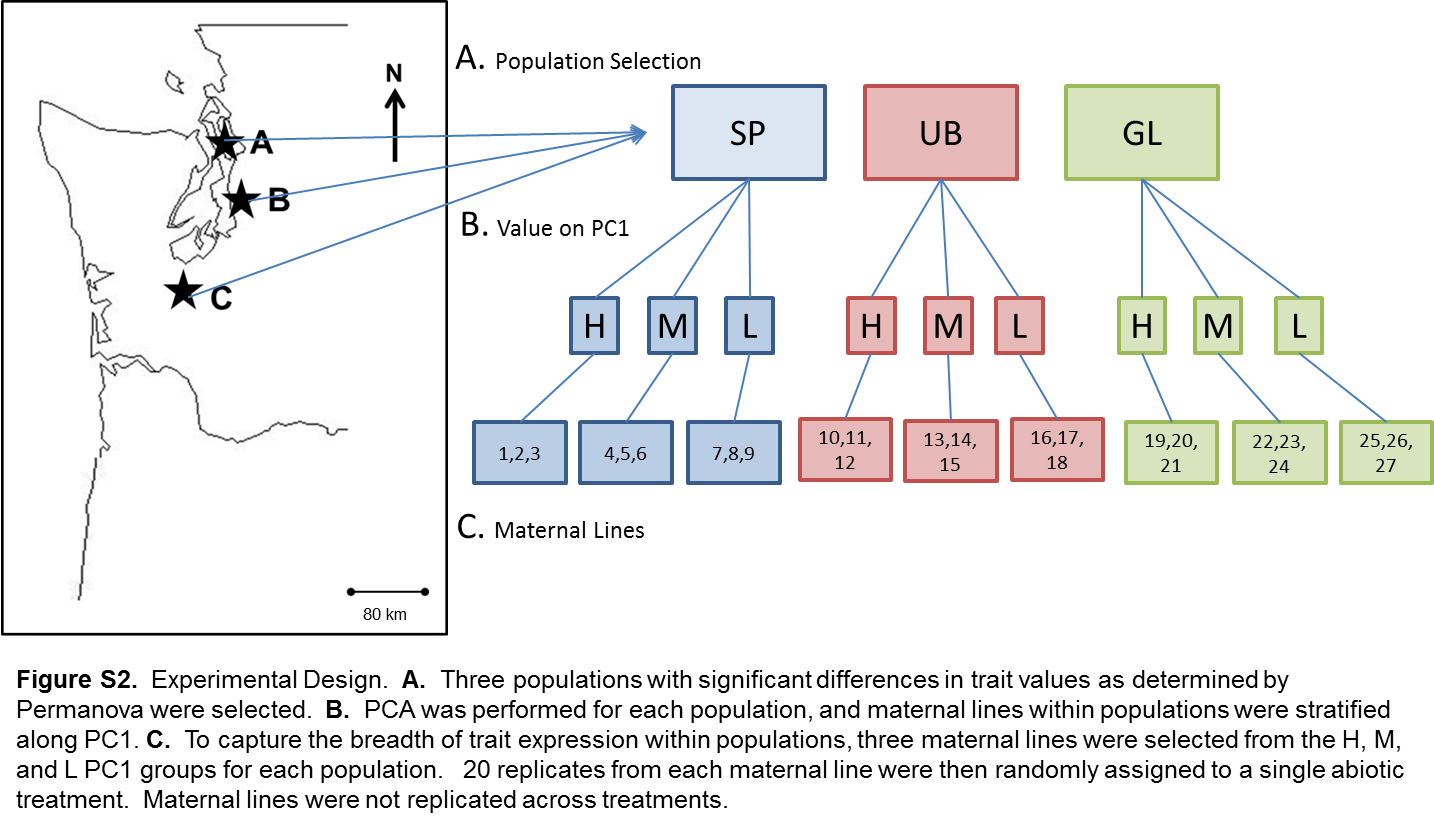

Supplement: Figure S1 — Diagram illustrating selection of populations and maternal lines. (DOCX) [file pone.0109870.s001.docx]
