# Supplementary material for: Intraspecific Trait Variation Driven by Plasticity and Ontogeny in Hypochaeris radicata
Source: PLoS One. 2014 Oct 21;9(10):e109870. doi: 10.1371/journal.pone.0109870 (PMC4204820; doi:10.1371/journal.pone.0109870)
Supplement: Figure S2 — Correlation Matrix of Traits. (DOCX) [file pone.0109870.s002.docx]

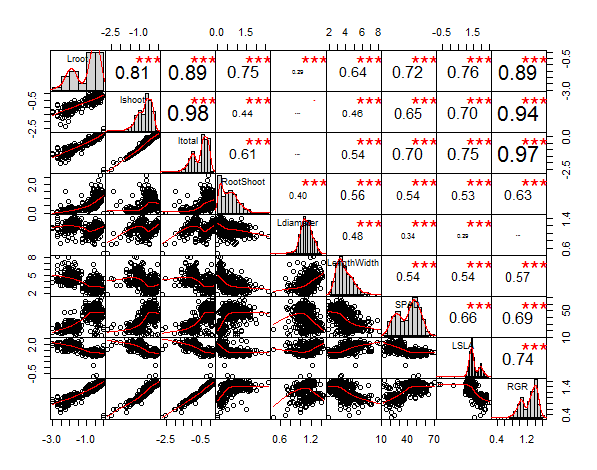


**Figure S1**. Correlation matrix of trait measurements. Bottom left displays scatterplot of data. Center diagonal displays variable name and histogram with kernel density overlay in red. Upper right displays correlation coefficient, with the associated *P*-value indicated with an asterisks (* = 0.05, ** = 0.01, *** = 0.001). Five traits were selected for analysis: diameter, leaf shape, root:shoot ratio, SLA, and final relative chlorophyll content
