# Supplementary material for: Intraspecific Trait Variation Driven by Plasticity and Ontogeny in Hypochaeris radicata
Source: PLoS One. 2014 Oct 21;9(10):e109870. doi: 10.1371/journal.pone.0109870 (PMC4204820; doi:10.1371/journal.pone.0109870)
Supplement: Table S1 — Table of Abiotic Conditions at Sample Locations. (DOCX) [file pone.0109870.s003.docx]

Table S1. Average annual rainfall, land-use history, and soil type for the collection sites of the three study populations.

| Population | Rainfall (cm) | Land Use | Soil Type |
| --- | --- | --- | --- |
| Glacial Heritage (GL) | 129 | Tilled Agriculture | Glacial Outwash |
| Smith Prairie (SP) | 65 | Agriculture | Glacial Outwash |
| Union Bay Natural Area (UB) | 97 | Reclaimed wetland | Mixed Urban Soil |
