# Supplementary material for: Intraspecific Trait Variation Driven by Plasticity and Ontogeny in Hypochaeris radicata
Source: PLoS One. 2014 Oct 21;9(10):e109870. doi: 10.1371/journal.pone.0109870 (PMC4204820; doi:10.1371/journal.pone.0109870)
Supplement: Table S2 — Results of General Linear Mixed Models. (DOCX) [file pone.0109870.s004.docx]

**Table S2.** Results and confidence intervals for each trait and each treatment as estimated with GLMM.

| Trait | Ambient | | | Drought | | | Shade | | |
| --- | --- | --- | --- | --- | --- | --- | --- | --- | --- |
|  | Estimate | Lower | Upper | Estimate | Lower | Upper | Estimate | Lower | Upper |
| Diameter | 13.8 | 12.1 | 15.9 | 10.4 | 9.1 | 11.9 | 15.4 | 13.4 | 17.7 |
| Leaf Shape | 3.3 | 2.8 | 3.7 | 3.2 | 2.7 | 3.7 | 4.8 | 4.3 | 5.3 |
| Root:Shoot | 0.8 | 0.7 | 0.9 | 0.8 | 0.7 | 1.0 | 0.2 | 0.1 | 0.3 |
| SLA | 18.6 | 17.0 | 20.5 | 21.3 | 19.4 | 23.3 | 78.4 | 71.4 | 85.9 |
| Chlorophyll Content | 46.7 | 44.6 | 48.8 | 46.8 | 44.7 | 48.9 | 25.6 | 23.5 | 27.7 |
